# Muscle Activity and Inactivity Periods during Normal Daily Life

**DOI:** 10.1371/journal.pone.0052228

**Published:** 2013-01-18

**Authors:** Olli Tikkanen, Piia Haakana, Arto J. Pesola, Keijo Häkkinen, Timo Rantalainen, Marko Havu, Teemu Pullinen, Taija Finni

**Affiliations:** 1 Neuromuscular Research Center, Department of Biology of Physical Activity, University of Jyväskylä, Jyväskylä, Finland; 2 Department of Health Sciences, University of Jyväskylä, Jyväskylä, Finland; Pennington Biomed Research Center, United States of America

## Abstract

Recent findings suggest that not only the lack of physical activity, but also prolonged times of sedentary behaviour where major locomotor muscles are inactive, significantly increase the risk of chronic diseases. The purpose of this study was to provide details of quadriceps and hamstring muscle inactivity and activity during normal daily life of ordinary people. Eighty-four volunteers (44 females, 40 males, 44.1±17.3 years, 172.3±6.1 cm, 70.1±10.2 kg) were measured during normal daily life using shorts measuring muscle electromyographic (EMG) activity (recording time 11.3±2.0 hours). EMG was normalized to isometric MVC (EMG_MVC_) during knee flexion and extension, and inactivity threshold of each muscle group was defined as 90% of EMG activity during standing (2.5±1.7% of EMG_MVC_). During normal daily life the average EMG amplitude was 4.0±2.6% and average activity burst amplitude was 5.8±3.4% of EMG_MVC_ (mean duration of 1.4±1.4 s) which is below the EMG level required for walking (5 km/h corresponding to EMG level of about 10% of EMG_MVC_). Using the proposed individual inactivity threshold, thigh muscles were inactive 67.5±11.9% of the total recording time and the longest inactivity periods lasted for 13.9±7.3 min (2.5–38.3 min). Women had more activity bursts and spent more time at intensities above 40% EMG_MVC_ than men (p<0.05). In conclusion, during normal daily life the locomotor muscles are inactive about 7.5 hours, and only a small fraction of muscle's maximal voluntary activation capacity is used averaging only 4% of the maximal recruitment of the thigh muscles. Some daily non-exercise activities such as stair climbing produce much higher muscle activity levels than brisk walking, and replacing sitting by standing can considerably increase cumulative daily muscle activity.

## Introduction

Recent epidemiological findings suggest that not only the lack of physical activity, but also prolonged times of sedentary behaviour, particularly sitting, increase significantly the risk of chronic diseases. This association persists even if people participate in moderate-to-vigorous intensity physical activities [1]–[9]. Since the continuum from total inactivity to vigorous-intensity training contains different physiological actions, one probably needs to meet a certain portion of different parts of this continuum for optimal health and fitness [10].

In the normal physical activity level (PAL) range, the distribution of time spent on activities with low and moderate intensity determines the daily activity level. High activity does not have much impact on PAL in the normal population because people spend more time sleeping and performing sedentary and light activities and hence the contribution from short term high intensity activity is small [11]. Consequently, an accurate estimation of time spent at low intensities is especially important during long term physical activity measurements [12]. It is also important to measure even short periods of physical activity accurately as even 15 min per day or 90 min a week provides a reduction in all-cause and all-cancer mortality [13].

Accelerometers and heart rate monitors are used extensively in research and can estimate energy expenditure, but cannot give any specific information about muscle activity. A major reason for limited data about the time spent in activities at different intensities is the difficulty to measure sitting time and patterns of spontaneous non-exercise movements [14]. Because every movement is generated by the muscle, the most direct approach to characterize daily physical activity is to measure electromyographic (EMG) activity. EMG can instantaneously detect the muscle activity burst durations and intensities. The muscle activity measurements present a novel avenue in physical activity measurements and some reports of daily EMG recordings have been published in humans (e.g., [15]–[19]). However, traditional EMG measurement devices set limitations to long ambulatory measurements in out-of-laboratory settings due to the fact that electrode placement and skin preparation require careful handling, and measurement systems include wires that need to be secured in order to avoid movement artefacts.

The development of clothes with embedded textile electrodes has made surface EMG measurements convenient even for whole day field measurements [16], [20]–[21]. For example, main locomotor muscle activity can be reliably measured using “EMG shorts” without skin preparation and problem of wires around the body, if one is only interested in the signal presence or absence and its relative amplitude [20].

In the present study the EMG shorts were used to assess muscle inactivity and activity periods during normal daily life of ordinary people. Muscle inactivity and different activity levels were categorized according to individually measured thresholds that were identified during functional tasks. From the methodological perspective the effect of different thresholds on inactivity and activity durations was tested.

## Methods

This study was part of a project “*Muscle loading during physical activity and normal daily life: correlates with health and well-being (EMG 24)*” that uses novel textile EMG electrodes embedded into shorts [20]. The study was approved by the ethics committee of the University of Jyväskylä and the subjects signed an informed consent prior to any measurements.

### Subjects

Subjects were recruited by advertisements to public places and different workplaces. We received a total of 245 contacts of which 122 were measured and meeting the inclusion criteria of being healthy with no major disorders or illnesses. Sufficient data was obtained from 84 subjects (20–76 years). Subject anthropometrics are presented in Table 1.

**Table 1:** Basic characteristics of the subjects.

Characteristics	Women (n = 44)		Men (n = 40)		All (n = 84)	
	Mean	SD	Mean	SD	Mean	SD
Age (years)	44.1	18.0	44.2	16.5	44.1	17.3
Height (cm)	166.0 ***	5.6	178.5 ***	6.6	172.3	6.1
Weight (kg)	62.3 ***	8.6	78.0 ***	11.9	70.1	10.2
BMI (kg/m^2^)	22.6 **	2.8	24.4 **	2.8	23.5	2.8
Knee extension MVC (kg)	93.0 **	36.2	123.5 **	47.2	107.0	44.0
Knee flexion MVC (kg)	59.9 ***	23.4	87.5 ***	33.6	73.0	32.0
VO_2_max (ml/kg/min)	41.0	10.9	44.3	12.5	42.4	11.6

Significant difference between genders are expressed as

** p<0.01;

*** p<0.001.

### Protocol

The protocol included assessments in the laboratory and physical activity measurements during normal daily life in the normal living environment of each individual. In laboratory, anthropometrics, questionnaires of physical activity and medical history were collected and subjects over 40 yrs were screened by a medical doctor. Further, the subjects performed the following tasks while hamstring and quadriceps muscle activity using EMG shorts was measured: lying down, standing, sitting, squatting, stair negotiation, walking, running and maximal isometric voluntary contraction (MVC).

#### Laboratory measurements

Body composition including fat percentage and visceral fat was measured with bioimpedance device (InBody 720, Biospace Ltd, Soul, Korea) in a fasting state. Resting metabolic rate (RMR) was measured for calculation of metabolic equivalent (MET). Subjects filled in activity and health questionnaires in which, for instance, their habitual physical activity, percentage of time spent sitting during working hours and health status were asked.

Subjects wore the shorts with textile electrodes (Myontec Ltd, Kuopio, Finland) during the activity laboratory test and performed bilateral maximal voluntary isometric contractions (MVC) in knee extension/flexion machine (David 220, David Health Solutions, Helsinki, Finland) with 140° knee angle in both flexion and extension. The EMG values from MVC were used to normalize all EMG data. In all MVC tests subjects were first familiarized with the testing actions. At least 3 trials of 3–5 seconds were performed (with 1 minute rest period between trials) in each test and if torque improved more than 5% in the last trial more trials were done. In all performances loud verbal encouragement was used to push subjects to their best.

On another day the subjects performed a special treadmill (OJK-1, Telineyhtymä, Kotka, Finland) test with 3 minute steps. All subjects started the test by walking at 4, 5, 6 and 7 km/h. The 5 km/h load was performed both at level and with 4° decline and incline. From this onwards, subjects who were under 29 years and also older subjects who were accustomed to run, performed one running load (10 km*h^−1^ for females and 12 km*h^−1^ for males). The next step for all participants was walking 5 km*h^−1^ with 8° ascent. After walking for 3 minutes with this load it was estimated how close participants were to their maximal oxygen consumption (VO_2max_). If two out of three of the following criteria were fulfilled participants continued with the same load until exhaustion: 1) VO_2max_ over 85% of estimated maximum, 2) heart rate over 90% of age estimated maximum, 3) Borg rating of perceived exertion over 16. If two out of three criteria were not fulfilled, participants continued the test by walking 7 km*h^−1^ with 10° ascent until exhaustion. This special protocol provided a possibility to measure heart rate and EMG in conditions that can occur during normal daily life (walking in different terrains).

#### Daily measurements

Measurements during normal daily life were performed either after the laboratory assessments or on separate days but not after the treadmill test. In the morning of each measurement day, the subjects came to the laboratory where the EMG recording devices were put on and set to record. Then they left for their normal living environments with instructions to live normal daily life as usual. To control the reliability of the EMG signal, the subjects were asked to perform and mark down to a diary special reference tests (15 seconds each) including lying down, standing and squatting preferably every 3 hours. The subjects were told to remove the equipment when taking a shower or swimming, or at latest before bedtime. When going to toilet, the subjects were instructed to carefully roll the shorts down and then roll the shorts back on. Measurement days were assigned for the subjects depending on their schedules, and some measurements were also done during weekends. During the measurement days, the subjects marked down the activities they have performed in ½ hour blocks, including sitting, standing, walking, bicycling and exercise for fitness, if any.

### EMG recordings

EMG was measured with shorts made of knitted fabric similar to elastic clothes used for sport activities or as functional underwear with the exception of capability to measure EMG from the skin surface of the quadriceps and hamstring muscles (Myontec Ltd, Kuopio and Suunto Ltd, Vantaa, Finland). To obtain the average rectified value of EMG (aEMG), the shorts were equipped with conductive electrodes and wires integrated into the fabric, which transfer the EMG signals from the electrodes to the electronics module. The textile electrodes are sewn onto the internal surface of shorts and consist of conductive yarns including silver fibres and non-conductive synthetic yarns woven together to form a fabric band. The electrodes are located such that the bipolar electrode pair lies on the distal part of the quadriceps and hamstrings, and the reference electrodes longitudinally at lateral sides (over tractus iliotibialis) on left and right side. Sizes of the electrodes were 2.5×9.5–14 cm for quadriceps muscles, 1.5×7.5–8 cm for hamstrings muscles and 2×29–33 cm for lateral grounding electrodes depending on the size of the shorts. In this study, five pairs of shorts (three different sizes) were used. Shorts were equipped with adjustable zipper in the waist and hems, and elastic Velcro straps in the hem for improved fit. Electrode paste (Redux Electrolyte Crème, Parker Inc., USA) was added on the electrode surfaces prior to every measurement day to improve and stabilize conductivity between the skin and electrodes. The subjects were also given a small bottle of the paste with them and instructed to add more paste after shower, for example. After every measurement day the shorts (electronics module detached) were washed with washing powder either by hand or in the washing machine.

The recording electronic module contains signal amplifiers, microprocessor with firmware, data memory and PC interface. In the module, the EMG signal is measured in its raw form with a sampling frequency of 1000 Hz and a frequency band 50 Hz–200 Hz (−3 dB). The raw EMG signal was first rectified and then averaged over non-overlapping 100 ms intervals. The averaged data was stored in the memory of the module from which the data was downloaded to a PC using the specifically designed HeiMo PC-software (Myontec Ltd, Kuopio, Finland) and then analyzed in MegaWin software (Mega Electronics Ltd., Kuopio, Finland).

### EMG analysis

#### Artefact correction

The entire EMG data was first visually assessed and corrected for artefacts. Use of automated algorithms was considered unreliable and difficult as data was not recorded in raw format but averaged over 100 ms intervals due to limited memory capacity of the module. Artefact removal and correction procedures were developed and refined with use of EMG shorts with online measurements possibility (Myontec Ltd, Kuopio, Finland). In separate laboratory testing session, different intentionally induced artefacts (i.e. movement of electrodes, module and lead wires; applying different degrees of pressure on electrode locations, placing shorts very close to electrical devices etc.) were compared to normal activities of daily living, and data was simultaneously screened on computer in real-time. With this procedure it was possible to educate research personnel responsible for artefact correction to differentiate between real muscle activity and artefacts with good degree of accuracy. The activity diaries and aEMG values during MVC (EMG_MVC_, see below) were compared to the signal and if non-physiological signals were observed the data were operated with four possible methods:

1. Brief high amplitude (>100%EMG_MVC_, <1 s) artefacts or ones which were identical and seen simultaneous on several channels. These artefacts seemed to be caused by electrical interference due to sharp movements of measurement module, lead wires or electrodes. These artefacts were replaced with mathematical interpolation from values prior to and after the artefact.
2. Continuing artefact occurring during obvious bilateral movement (i.e. walking) was corrected by copying the data from contralateral channel. These artefacts were noticed as considerably higher peak activities or considerably increased ‘baseline’ activity than on contralateral side which could not be explained by physiological factors. These artefacts seemed to be caused by extraneous movement of electrodes in relation to skin, or movement of tissues between electrodes and muscle (i.e. skin and fat tissue) due to impact forces of locomotion.
3. In case the artefact was longer than 30 min, the signal was removed from that particular channel; therefore the recording time can vary between the four channels.
4. In case the signal was consistently abnormal throughout the measurement day(s) the particular channel was removed fully from the analysis. These cases were probably caused by improper function of the measurement device, improper size of the shorts or impedance problems between skin and electrodes.

A total of 222 days were measured equalling 888 channels of daily data (4 channels per measured day; right quadriceps, right hamstring, left quadriceps, left hamstring), from those channels 23% were not included in the analysis because of non-usable data, leaving 684 channels of daily data for analysis. On those channels recording time was on average 11.3±2.0 hours and on average 22.9±50.4 min (4.1±9.9%) was corrected for artefacts with the methods explained above. On average correction method 1 was used for 1.3±5.5 min (0.2±1.5%) per channel, method 2 for 3.3±8.6 min (0.7±2.6%), and method 3 for 18.4±49.5 min (3.1±9.3%)

#### Baseline correction

Signal baseline was determined as moving 5 minute minimum. The minimum of the following 5 minute data window was subtracted from each data point. The 5 min window was tested to be the best one to correct for minor baseline fluctuations without affecting the actual signal amplitude, inactivity times or activity burst durations.

#### Data normalization

Maximal EMG values (EMG_MVC_) were taken as an average from a 1 s period during MVC. The EMG signal from each of the four muscles (right quadriceps, right hamstring, left quadriceps, left hamstring) was normalized to the EMG_MVC_ value in a given muscle, and the mean of the four channels is presented in the results as % of EMG_MVC_. Also the thresholds for light, moderate and vigorous intensities were determined channel-by-channel from normalized EMG before averaging the four channels.

#### Threshold level determinations

Threshold levels between different muscle activity intensities during normal daily life were based on the standing reference test and an incremental treadmill walking test. The threshold between inactivity and light-intensity activity was set as an EMG value corresponding to 90% of the EMG value of standing on each individual and each channel. Thus, inactivity in this report represents the true individual behaviour below standing activity. The thresholds between light and moderate, and moderate and vigorous intensity were defined as 3 METs (metabolic equivalent) and 6 METs, respectively. These thresholds were assessed from the incremental treadmill walking test in the following way: From each load EMG was taken as one minute average from the middle of the load and VO_2_ as an average from two last minutes of the load. VO_2_ values were transformed to METs by division with resting metabolic rate (RMR) [22]. EMG values from each load were plotted against the corresponding MET values, and the EMG values corresponding to 3 METs (threshold for moderate intensity) and 6 METs (threshold for vigorous intensity) were calculated by regression analysis and used as individual threshold values. In the regression, additional point was added for 1 MET = 0 EMG representing the resting state and the highest VO_2_ value was excluded since the normal daily activities are lower and the highest value could have biased the regression equation.

The artefact corrected and normalized EMG data was run through a custom made Matlab script (MATLAB, MathWorks, Massachusetts) where the following EMG variables were calculated:

#### Burst, inactivity and activity calculations

**Figure 1:**
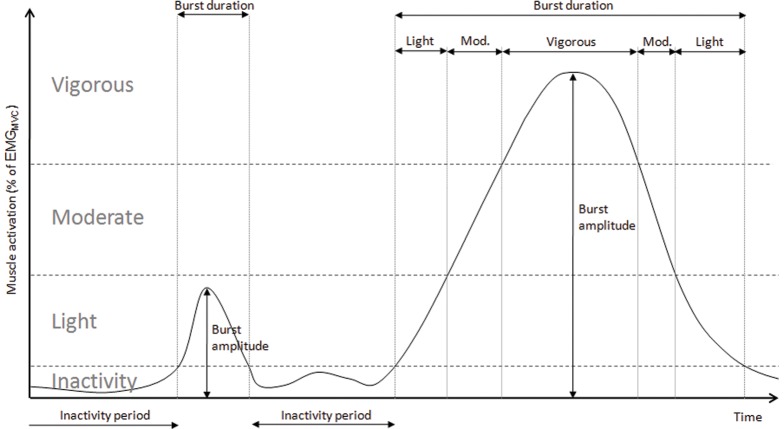
Analysis of burst characteristics and muscle activities. Schematic drawing depicting analysis of burst amplitude, duration, inactivity periods and differentiation to light, moderate and vigorous muscle activities. The thresholds for inactivity, light, moderate and vigorous activities were determined individually (see text).

Determination of burst variables is shown in Figure 1. The following variables were calculated:

average EMG from the entire recording period (% of EMG_MVC_)
total inactivity duration (min)
durations of five longest continuous inactivity periods (min)
light intensity activity time (min)
moderate intensity activity time (min)
vigorous intensity activity time (min)
number of bursts (#)
average duration of bursts (s)
average amplitude of all bursts (% of EMG_MVC_)
burst rate (bps)
total area of bursts (% of EMG_MVC_ * s)

#### Activity histograms

The distribution of muscle activity for different levels was calculated for different percentages of EMG_MVC_: 0–1, 1–2, 2–3, 3–4, 4–5, 0–5, 5–10, 10–20, 20–30, 30–40, 40–50, 50–60, 60–70, 70–80, 80–90, 90–100 and >100. After that the calculations were completed with the individual threshold levels for inactivity, light-, moderate- and vigorous-intensity activity.

#### Averaging of variables

After the variables (e.g. inactivity time, burst amplitude) were extracted from each four channels, they were averaged across different days within individual. Then, the different variables from the four muscle groups were averaged to get one descriptive variable for each subject, and these averaged values were used for further analysis.

#### Repeatability of data

Repeatability of the EMG data was assessed using the reference tests from 20 subjects during 1–3 days. The reference test consisted of lying down quietly for 15 s, standing quietly for 15 s and remaining in half-squat position for 15 s. From each task average EMG values from a 10 s period were analyzed. Intraclass correlation was calculated between aEMG values of two tests within the same day. The mean intraclass correlation of all four channels was 0.73±0.19 in lying down, 0.75±0.16 during standing and 0.73±0.09 during half-squat. Day-to-day intraclass correlations of all four channels were 0.80±0.37 in lying down, 0.97±0.03 during standing and 0.90±0.07 during half-squat. It should be noted that these analyses were done before signal baseline correction, which can explain the higher day-to-day than within-day repeatability

#### Statistical analyses

Statistical analyses were performed with PASW Statistics v.18.0 (SPSS inc. Chicago, Illinois, US). Data is presented as mean ± standard deviation. The differences between the genders were studied with independent samples Mann-Whitney U-test after checking distribution of the data with Saphiro-Wilk test. Significance level was set at P<0.05.

## Results

**Figure 2:**
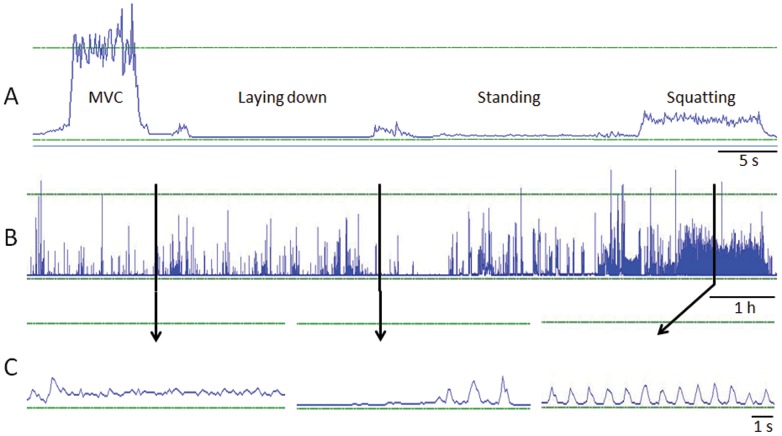
Example EMG data from laboratory and field measurements. Examples of averaged EMG data of left quadriceps femoris from one subject. Part A shows from the laboratory measurement session MVC of knee extension, lying down, standing still and squatting. Part B shows EMG activity during the entire day. Part C shows zoomed areas from daily EMG data (thick vertical lines show which parts of the data are zoomed). Horizontal lines represent baseline and 100% of EMG_MVC_.

**Figure 3:**
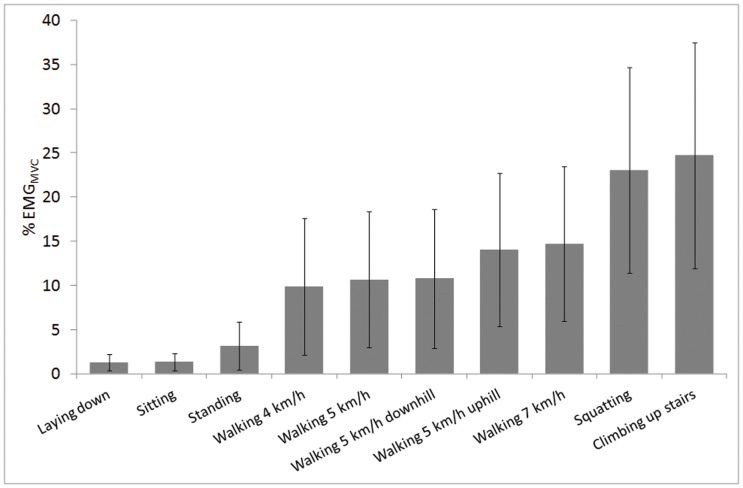
Average muscle activity in daily tasks. Average (±SD) quadriceps and hamstring muscle activity for different daily tasks measured in the laboratory (n = 63–84).

Examples of raw recordings during the reference tests (Fig. 2A) and during normal daily life (Fig. 2B, 2C) are shown in Figure 2. Muscle activities during the different functional tasks in the laboratory are shown in Figure 3. For example, muscle activity during standing is almost 2.5 times higher than during sitting. Downhill walking yields similar average muscle activation as walking on level ground and muscle activity is increased on average 30.8% in 4° uphill compared to level ground with same walking speed (5 km/h). While stairs ascend is typically considered as non-exercise activity it produces EMG amplitudes over 20% of EMG_MVC_ that are much higher than in brisk walking.

The individually defined inactivity, moderate activity (3 MET) and vigorous activity (6 MET) thresholds are shown in Table 2, while Table 3 compares total daily activity and inactivity times calculated using the individual inactivity thresholds and absolute thresholds of 1, 2 and 5% of EMG_MVC_. On average, the 2% EMG_MVC_ threshold yields activity and inactivity times similar to the individually determined threshold.

**Table 2:** Average EMG activities at different thresholds that were defined from the standing and incremental treadmill walking test.

Laboratory tests	Women (n = 44)		Men (n = 40)		All (n = 84)	
	Mean	SD	Mean	SD	Mean	SD
Inactivity threshold = 0,9×standing (% EMG_MVC_)	2.5	2.0	2.4	1.4	2.5	1.7
Moderate activity threshold (% EMG_MVC_)	6.9	4.7	5.6	2.0	6.3	3.7
Vigorous activity threshold (% EMG_MVC_)	15.4	11.2	12.9	5.0	14.2	8.8

**Table 3:** Inactivity times during normal daily life based on different thresholds.

Inactivity threshold	Inactivity time (% of total time)	
	Mean	SD
90% of EMG of standing	67.5	11.9
1% EMG_MVC_	54.5	17.1
2% EMG_MVC_	68.2	15.4
5% EMG_MVC_	81.4	11.9

The first row represents results using individually determined threshold below standing activity (mean of four muscle groups) that is compared to the absolute % levels of each individual's EMG amplitude during maximal voluntary contraction (EMG_MVC_).

During normal daily life, the average EMG amplitude was 4.0% of EMG_MVC_, and average burst amplitude was 5.8% of EMG_MVC_ (Table 4). The subjects had 12,600±4,000 muscle activity bursts during a day and burst duration was 1.4±1.4 s. Women had significantly more (15.7%, P<0.05) activity bursts than men and time over 50% EMG_MVC_ was 73% longer than in men (p<0.05). Overall, women spent more time at intensities above 40% EMG_MVC_ compared to men (p<0.05) (Table 5).

**Table 4:** EMG volume and rate indicators from normal daily life for women and men presented relative to isometric MVC (±SD).

	Women (n = 44)		Men (n = 40)		All (n = 84)	
	Mean	SD	Mean	SD	Mean	SD
**EMG volume indicators**						
Average amplitude (% EMG_MVC_)	4.4	3.0	3.5	2.0	4.0	2.6
Average burst amplitude (% EMG_MVC_)	5.9	3.7	5.7	3.1	5.8	3.4
Total area (% EMG_MVC_ * s)	148 885	89 116	116 089	73 100	133 268	83 046
Time over 50% EMG_MVC_ (min)	6.4*	7.1	3.7*	5.4	5.1	6.4
Activity time (min)	229	86	200	84	215	86
**EMG rate indicators**						
Number of bursts	13 410*	3 549	11 591*	4 331	12 544	4 021
Average burst duration (s)	1.4	1.3	1.5	1.5	1.4	1.4
Burst rate (bps)	0.41	0.15	0.37	0.14	0.39	0.15

Significant difference between genders are expressed as

* p<0.05.

**Table 5:** Time spent at different EMG intensities (relative to isometric MVC) in normal daily life for women and men.

	Women (n = 44)		Men (n = 40)		All (n = 84)		All (n = 84)	
	Mean	SD	Mean	SD	Mean	SD	Mean	SD
EMG_MVC_: time (%)							Time (mm∶ss)	
0–1%	53.3	18.6	55.9	15.3	54.5	17.0	361∶39	126∶20
1–2%	13.0	6.1	14.3	8.6	13.6	7.3	91∶11	51∶32
2–3%	6.6	3.7	6.2	4.1	6.4	3.9	42∶48	27∶54
3–4%	4.4	2.8	3.6	2.2	4.0	2.5	26∶37	17∶15
4–5%	2.9	1.4	2.7	1.7	2.8	1.5	18∶51	10∶17
0–5%	80.2	13.1	82.8	10.5	81.4	11.9	541∶06	120∶08
5–10%	8.3	5.0	8.0	6.0	8.2	5.5	53∶44	34∶19
10–20%	6.1	3.6	5.4	3.1	5.8	3.4	38∶10	21∶53
20–30%	2.5	2.1	2.0	1.6	2.2	1.9	14∶40	11∶28
30–40%	1.3	1.3	0.9	0.8	1.1	1.1	07∶01	06∶30
40–50%	0.7*	0.7	0.4*	0.4	0.6	0.6	03∶53	03∶40
50–60%	0.4*	0.4	0.2*	0.2	0.3	0.4	01∶59	02∶14
60–70%	0.2*	0.3	0.1*	0.2	0.2	0.2	01∶07	01∶23
70–80%	0.1*	0.2	0.1*	0.1	0.1	0.1	00∶40	00∶53
80–90%	0.1*	0.1	0.0*	0.1	0.1	0.1	00∶25	00∶36
90–100%	0.1*	0.1	0.0*	0.1	0.0	0.1	00∶16	00∶25
≥100%	0.1*	0.2	0.1*	0.2	0.1	0.2	00∶38	01∶16

Time expressed as percentage from total measurement time and as minutes and seconds.

Significant difference between genders are expressed as

* p<0.05.

Table 5 shows the time spent at different EMG intensities in detail. 97% of measurement time muscle activity was below 30% of EMG_MVC_ and less than 2 min was at intensities above 70% EMG_MVC_. Accumulated time over 50% of EMG_MVC_ was only 5.1±6.4 min (range 0.0–21.2 min).

**Figure 4:**
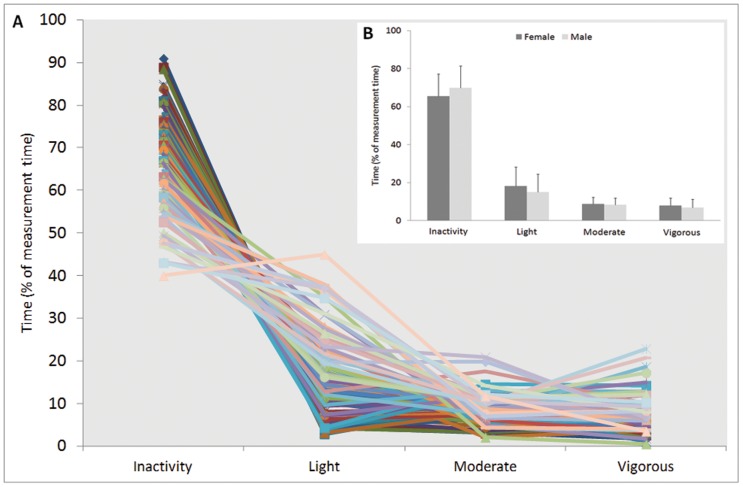
Muscle activity levels during normal daily life. A) Time spent at different muscle activity levels during normal daily life based on individual threshold values (2.5±1.7, 6.3±3.7 and 14.2±8.8% EMG_MVC_ for inactivity, moderate activity and vigorous activity thresholds, respectively). Each line represents one individual (n = 84). B) Duration of mean (±SD) quadriceps and hamstring muscle activity at different intensities did not differ between males (n = 40) and females (n = 44) during an 11 hour measurement.

Thigh muscles were inactive for 67.5±11.9% of the measurement time (Fig. 4). Corresponding times for light, moderate and vigorous activity were 16.7±9.9%, 8.5±3.4% and 7.3±4.2%, respectively. Inactivity ranged from 39.9 to 90.9% of measurement time highlighting large inter-individual differences in physical activity behaviour. Inter-individual variance was considerably high also in light, moderate and vigorous activities ranging from 2.8 to 45.0%, 2.0 to 20.9% and from 0.5 to 22.8% of measurement time, respectively.

The longest continuous inactivity periods lasted for 13.9±7.3 min (range 2.5–38.3 min). 2^nd^, 3^rd^, 4^th^ and 5^th^ longest inactivity periods lasted 9.8±4.5 min, 8.0±3.4 min, 7.0±3.0 min, 6.3±2.7 min, respectively. Bivariate correlations revealed that the longest inactivity period was associated with the 2^nd^ (R^2^ = 0.80), 3^rd^ (R^2^ = 0.72), 4^th^ (R^2^ = 0.70) and 5^th^ (R^2^ = 0.68) longest inactivity periods (P<.001). Thus the longest inactivity period represents the trend of the lengths of other inactivity periods, too.

## Discussion

This study reports EMG levels during normal daily living and provides reference points measured in the laboratory during simulated daily activities. The main finding of this study was that thigh muscles are inactive over 65% of the measurement time and only a fraction of muscle's maximal voluntary strength capacity is used during normal daily life. Average EMG amplitude was 4% EMG_MVC_ which is below the mean EMG level required for walking (Fig. 3). In our study, quadriceps and hamstring muscles were active on average 34% of total time (3.6 hours from 11 hour recordings), while others studies have reported lower values for EMG duration for vastus lateralis during 8 to 15-h recordings, including 13% [18], 12% [27], 10% [17], and 9% [28]. One factor possibly explaining differences in activity times is that in our study, with a comprehensive sample size, we also measured hamstrings in addition to quadriceps, and instead of single muscles whole muscle groups were measured. Also many other factors such as differing subject group, measurement systems, thresholds employed and analysis procedures used, probably contribute to the differences in activity times between the studies.

### Muscle activity during normal daily life

In the present study the mean EMG burst amplitude of quadriceps and hamstring muscles was 5.8% of EMG_MVC_ that is comparable to that found by Klein et al. [18] where mean vastus lateralis EMG was 6.9% EMG_MVC_. On the other hand, Kern et al. [17] reported mean burst amplitude of 17% MVC (for vastus lateralis) and 18% MVC (for vastus medialis) but these subjects were moderately active college students who are likely to be more active than the present group with large age range. In addition, Kern et al. [17] used burst threshold of 2% of MVC which may have left some smaller bursts out of the analysis whereas the numbers by Klein et al. [18] are based on values above a baseline, which may explain the smaller mean amplitudes.

As average EMG amplitude was only 4% EMG_MVC_, the daily muscle activity could be cumulated by walking at 7 km/h for 3 hours or ascending stairs for less than 2 hours. Fifteen subjects had average daily EMG amplitude under 2% of EMG_MVC_ which could be achieved by less than 1 hour of stair climbing or about 1.5 hours of walking at 7 km/h. These subjects could have increased their thigh muscle activity 50% by standing 3.5 hours more during the day.

On average, the present subjects had muscle activity over 90% of EMG_MVC_ only for 56 seconds (Table 5). Thirty-six subjects had less than 10 seconds of muscle activity over 90% EMG_MVC_ and 39 subjects had less than on minute of muscle activity over 60% EMG_MVC_. Larger muscles, such as those in leg or arm are reported to recruit motor units at least up to 90% of MVC [23]. Specifically, in isometric conditions vastus lateralis muscle maximum recruitment threshold has been shown to be 95% of MVC [24] although in rapid contractions the threshold is lower [25]. Therefore, some motor units may have very little if any activity during the daily life in these subjects. Long-term consequences of limited activity of the highest threshold motor units is uncertain, but it is conceivable that such a condition for many years could contribute to age-related weakness and muscle atrophy [18]. To express the activity times from another perspective, in hypertrophic strength training typical activity duration for one single exercise is about 60–220 seconds (4–6 sets with 8–12 repetitions each lasting 2–3 seconds) and the intensity level varies between 70–85% of one repetition maximum. This kind of hypertrophic training usually includes 2–3 different exercises for one muscle group leading to increase in muscle mass. While the maintenance of the acquired muscle mass and strength after an intensive training period requires 1–1.5 training sessions per week, it is not quite clear how much and what type of muscle activity is needed to maintain muscle mass in normal subjects during daily life [26].

A very large variability between subjects in total inactivity times ranging from 40 to 91% of total time were found (Fig. 4). Also light activity (3–45% of total time) has much higher between-subjects variability than moderate (2–21% of total time) or vigorous (1–23% of total time) activity indicating that individual differences in light physical activity have greater influence on inactivity time than moderate or vigorous physical activity. It is important to measure also short periods of physical activity accurately as even 15 min of physical activity per day provides a reduction in all-cause mortality and extends an individual's lifespan for an average of 3 years [13].

### Continuous inactivity periods

There was a very large variation between subjects in continuous inactivity periods. One subject did not have inactivity periods longer than 3 minutes and in the other extreme one subject had the longest inactivity period lasting almost 40 minutes and even his 5^th^ longest inactivity period was over 15 minutes. It should be reminded that activity burst as short as 0.1 second breaks the inactivity period reported in this paper. Therefore, it is possible that a long inactivity period is stopped by very short activity that could be done during sitting followed by another long inactivity period. As our inactivity threshold was individually determined based on a functional task it is highly likely that these values represent the true behaviour of the subjects. Short inactivity periods may reflect a behaviour where person is rarely sitting or sitting but fidgeting legs very often. At this point we do not know how long the activity period should be to be physiologically significant or how long inactivity can be sustained without consequences to health although a recent report shows adverse effects of 1 day sitting on metabolism [29].

### Differences between males and females

Women had more activity time 40–100 of EMG_MVC_, over 50% EMG_MVC_ and more activity bursts than men. Kern et al. [17] reported that women had 70% higher summed duration of bursts in biceps brachii than males and their small sample size might have hindered differences in other muscles. Finnish women have been reported to take more steps during the day than Finnish men [30]. Shorter stature of females could affect to higher burst count as shorter persons need to take more steps to cover the same distance in walking or running. Women had significantly lower torques in MVC meaning that they need to use higher relative force for the same absolute tasks than men, possibly explaining women having more activities at higher intensity.

### Inactivity threshold determination

We decided to use individual task-based threshold because of the large variability in MVC torques and EMG_MVC_ values. If we had, for example, used 1.7±0.2% of EMG_MVC_ (determined from the baseline) as inactivity threshold as used by Klein et al. [18], some of the strongest subjects would not have exceeded the threshold during standing, which would then had been mistakenly classified as inactivity. Indeed, we used quiet standing as a reference activity as it can be considered as the lowest level of activity in daily life. Inactivity threshold was set to 90% of activity of standing to ensure that standing would be classified as activity in all cases. Individual inactivity threshold is especially important when subjects differ largely in their force production capabilities, as in the present heterogeneous study group.

The individually determined threshold (2.5±1.7% EMG_MVC_) yielded approximately the same inactivity times as 2% EMG_MVC_ threshold, but with smaller variation in inactivity times (SD 11.9 and 15.4, respectively). Inactivity threshold of 1% EMG_MVC_ decreased the inactivity time considerably, and threshold of 5% EMG_MVC_ increased it. Therefore, our results support the suggestion from Klein et al. [18] that even a small change in the threshold may result in a very large change in the daily EMG duration.

### Maximal voluntary muscle force and EMG activity measurement

In addition to the between subject variability in MVC and EMG_MVC_ values, the method by which these values are measured is important when comparing different studies. The maximal voluntary muscle force and muscle activity is a critical measurement as changes in the threshold (%EMG_MVC_) affect the inactivity time and time spent in different activity levels. Klein et al. [18] normalized their data with EMG_MVC_ values measured in 80° knee angle, while we used 140° knee angle (knee fully extended is 180°). Although 120° is reported to be optimal for maximal force production [31] we wanted to use a knee angle that is closer to ones used commonly in normal daily life. Careful attention was paid to ensure that real maximum was achieved in MVC testing by doing enough trials and by using loud verbal encouragement.

### Methodological limitations

Even though considerable amount of data (23%) had to be excluded from the analysis, sample size (n = 84) and recording time (over 10 hours/day) remained relatively good. The cross-sectional design, however, adds variability to our study as people are likely to be more physically active in summer than in winter [32]–[34] and in weekends than weekdays [33]. On the other hand, light physical activities dominate over moderate activities on weekdays [30]. In our study we only measured muscle activity from thigh muscles. Probably thigh muscle activity gives a good indication of activity of other leg muscles also as a major portion of daily physical activity consists of standing and locomotion [11]. We have tested that thigh muscle activity correlates highly with energy expenditure in walking at different speeds in level and in uphill and downhill (unpublished results).

### Representativeness of subject group

Subjects were recruited through advertisements in public places and different workplaces and therefore do not represent a randomly selected sample. Often more active people are more interested taking part in research projects which include physical activity assessments and although this bias cannot be excluded, our results do show that we also had inactive and sedentary subjects in our study. This study was conducted with Finnish people living in a rather small city with good possibilities to walk or bicycle to work. However, the nature of office work does not differ considerably between cultures and therefore our data gives insight into inactivity of modern societies.

### Relevance of the study

Wearable electromyography enables measurement of details of muscle inactivity and activity during normal daily life. The muscle activity patterns reported in this paper are significant for understanding intensity, amount and distribution of physical activity which is typical in healthy adults. These data can be used as a reference point of activity levels and patterns that maintain or can recover normal muscle properties. Further, the data can be valuable for future interpretations of activity patterns in different disorders affecting locomotor abilities, motor dysfunctions, or overall physical activity.
